# Feasibility of electrochemical impedance spectroscopy for *in situ* detection of water stress in plants

**DOI:** 10.2478/joeb-2026-0003

**Published:** 2026-03-17

**Authors:** Rintaro Shinoda, Mutsumi Sugiyama

**Affiliations:** Faculty of Science and Technology, Tokyo University of Science, 2641 Yamazaki, Noda, Chiba 278-8510, Japan

**Keywords:** Electrochemical impedance spectroscopy, plant water stress, bioimpedance, equivalent circuit analysis, non-invasive plant monitoring

## Abstract

Electrochemical impedance spectroscopy (EIS) has been widely applied to bioimpedance measurements in human and animal systems; however, its potential for direct plant monitoring remains less explored. This study uses *Komatsuna* (Brassica rapa) to examine the feasibility of using EIS for *in situ* detection of plant water stress.

Impedance spectra are measured noninvasively and analyzed using an equivalent circuit model designed to separate plant-related electrical properties from the electrode–plant interface. Changes in the low-frequency impedance region were observed under both irrigation and drying conditions, while the high-frequency response remained relatively stable. In particular, variations in the extracellular resistance parameter (*R_o_*) preceded visible water-stress symptoms and continued even after visual changes became indistinguishable. Although the number of tested plants was limited, these results suggested the potential of EIS as a rapid and cost-effective tool for early, *in situ* assessment of plant water status. The present study provides a proof-of-concept for extending bioimpedance-based approaches to plant systems, with implications for precision agriculture and plant physiology research.

## Introduction

Recently, the research interest in “smart agriculture”, which uses advanced technology to automate and improve farming efficiency, has significantly increased. In such systems, a wide variety of data are collected by monitoring the plant cultivation environment, including soil water content [1,2], temperature [3], and CO_2_ concentration [4], using Internet of Things (IoT) and information and communication technology (ICT) devices to optimize cultivation management [5]. These approaches provide valuable environmental information, but do not directly monitor the physiological state of plants. Therefore, direct plant monitoring is essential to bridge this gap and enable adaptive cultivation management.

Conventional direct plant monitoring methods can be divided into techniques that evaluate external or internal plant conditions. External monitoring methods based on plant shape and color [6], are effective for detecting visible stress symptoms such as wilting and leaf discoloration. However, physiological disturbances often occur prior to visible changes, resulting in a time lag that can lead to irreversible damage. In contrast, methods targeting internal conditions, including transpiration flow [7] and chlorophyll fluorescence [8], enable earlier detection of stress responses but require complex and expensive instrumentation, limiting their practical use.

Electrochemical impedance spectroscopy (EIS) has been widely applied in biomedical and animal studies, where impedance responses reflect tissue structure, hydration state, and ionic transport processes [9,10,11]. In contrast, impedance-based investigations of intact plants remain relatively limited, despite the central role of water and ion dynamics in plant physiology. Plant tissues possess unique structural and physiological features, such as rigid cell walls, large vacuoles, and dynamically regulated extracellular spaces, which highlight the importance of plant-specific impedance studies rather than direct extrapolation from animal systems. To address these challenges, we propose EIS as a practical and noninvasive approach for direct [12,13,14] and real-time [15] monitoring of plant physiological conditions. Unlike conventional direct current (DC) measurements, EIS employs alternating current (AC) to probe frequency-dependent electrical properties, enabling separation of contributions from cell membranes, intracellular fluids, and extracellular fluids. EIS has been extensively used in chemical materials and semiconductor research [9,10,11,16,17,18,19,20,21,22,23], and its application to plants offers a multifaceted view of physiological states through electrical decomposition.

Previous electrical studies of plant water status have often relied on invasive electrode insertion and have primarily focused on impedance magnitude, without fully exploiting equivalent circuit modeling [24,25,26]. Moreover, impedance contributions from the electrode–plant interface are frequently neglected, limiting the physiological and electrical realism of such analyses. Therefore, this study aims to improve the evaluation of plant water status using EIS by explicitly separating plant-related impedance components from non-plant contributions. Using *Komatsuna* (Brassica rapa) as a model system, we investigated water stress, a key factor influencing plant growth and crop quality [27]. The results suggest that EIS enables noninvasive detection of water-status-dependent impedance changes, including early stress stages that precede visible symptoms [28]. These findings highlight the potential of EIS as a cost-effective and physiologically informative tool for monitoring plant water stress and supporting precision agriculture.

## Experimental methods

### Ethical approval

The research conducted is not related to either human or animal use.

### Selection of plants and EIS measurement method

**Fig. 1.**
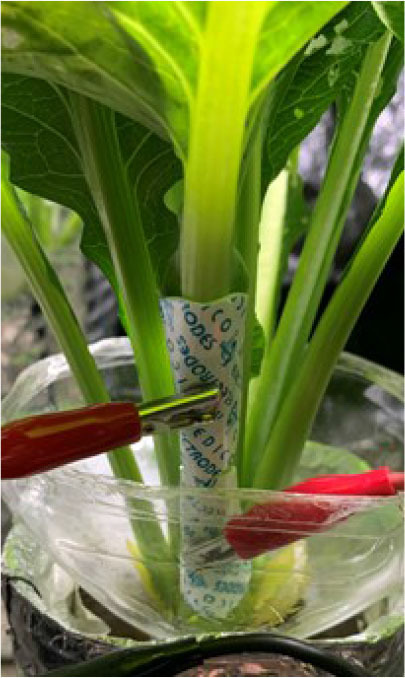
Experimental setup for EIS measurements. Two ECG electrodes were noninvasively attached to the petiole of a *Komatsuna* plant with an electrode separation of approximately 25 mm, enabling *in situ* impedance measurements without tissue penetration.

For the EIS measurements, the *Komatsuna* (Brassica rapa L. Perviridis, Variety: Hamatsuzuki, Japan) was selected because its appearance changes easily in response to water stress, making it suitable for monitoring. *Komatsuna* is also widely used in controlled cultivation experiments, making it an appropriate model plant for feasibility-oriented methodological studies. The experimental setup for the EIS measurements is illustrated in Fig. 1. Two gel-type electro-cardiogram (ECG) electrodes (MSGLT-08, Medico, Japan) with a circular contact area (radius: 4 mm, effective area: 50.2 mm^2^) were attached to the petiole of the *Komatsuna* plant with a fixed inter-electrode distance of approximately 25 mm. These electrodes allow noninvasive EIS measurements by adhering to the plant's surface without penetration. In addition, the electrodes were kept fixed on the plant throughout the experiments to minimize variability in electrode geometry and contact conditions. Under the present experimental conditions, no noticeable drying of the electrode gel was observed even during continuous measurements conducted over several days. All measurements were performed in a controlled greenhouse environment, where temperature and illumination were maintained as constant as possible. A frequency response analyzer (IM3533-01, HIOKI, Japan) was used to conduct the measurements.

In all EIS measurements, a 1 V AC signal was applied to the *Komatsuna* using the two-terminal method. This voltage amplitude was selected based on preliminary tests showing that lower voltages resulted in increased noise. In contrast, higher voltages provided no substantial improvement in signal quality, consistent with previous studies demonstrating that 1 V AC excitation does not induce observable physiological or structural damage to plant tissues under similar conditions [29]. No apparent nonlinear effects or visible changes were observed during repeated measurements. In a single frequency sweep, 100 data points were recorded over the range from 10 Hz to 200 kHz. The frequency range was selected based on preliminary test measurements conducted prior to the main experiments.

In these tests, the overall shape of the Nyquist plots was carefully inspected to ensure reliable identification of the low-frequency semicircle. At excessively high frequencies, meaningful plant-related impedance information became unreliable, whereas at lower frequency limits, the semicircle apex was insufficiently resolved. Although the upper frequency limit does not provide an ideal estimate of all parameters, it was sufficient for the stable extraction of the fitted data. Simpler equivalent circuit models were also examined, but the present model provided the most stable fitting behavior across different plants and conditions [12,13,14,15,29]. To improve measurement reliability and suppress random noise, 10 consecutive frequency sweeps were performed and averaged at each measurement time point, and the measurements were repeated every 2 h. The averaged impedance response at each time point was subsequently used for analysis, providing a more reliable dataset despite the limited number of biological replicates. The applied voltage did not significantly affect the cell tissues [12,13,14,15].

### Selection of plants and EIS measurement method

In this experiment, hydroponically grown *Komatsuna* plants were prepared to observe the recovery process resulting from water absorption. Hydroponic cultivation was adopted to ensure uniform initial water conditions and to minimize variability associated with soil heterogeneity. Nine independent *Komatsuna* plants were measured under the same time-resolved protocol. Two additional plants were prepared as controls under identical initial drying conditions, without irrigation, and were measured in parallel with the irrigated plants. Electrodes were attached to the petiole of each plant, and measurements were taken after a 2 h waiting period to allow stabilization of the electrode–plant interface. The experiment was conducted in a controlled greenhouse, with temperatures ranging from 22 °C to 24 °C. Temperature was monitored throughout the experiment, and no abrupt fluctuations were observed during the measurement period. One group (irrigated *Komatsuna*, 10 samples) was irrigated, whereas the other group (control *Komatsuna*, 2 samples) was kept dry. Although the number of control plants was limited, repeated time-resolved measurements were conducted for each individual to capture reproducible trends in impedance evolution. EIS measurements were conducted before irrigation and then every 2 h after irrigation, with appearance photographs taken at the same interval. Furthermore, the water content of the *Komatsuna* was measured by cutting the base of the petiole to determine the fresh and dry weights, from which the water content was calculated using the following formula:

(1) $$\text{Water}\;\text{content}=\frac{\text{fresh}\;\text{weight}-\text{dry}\;\text{weight}}{\text{fresh}\;\text{weight}}\times 100\%.$$

This destructive measurement was performed at selected time points and treated as a reference indicator of plant water status.

Drying monitoring experiments were conducted in a controlled greenhouse, with temperatures ranging from 29 °C to 31 °C. The higher temperature condition was selected to promote water loss and accelerate the drying process. To ensure comparable initial conditions, the *Komatsuna* plants were thoroughly hydrated prior to the experiments. Three independent plants were measured once each under the same time-resolved protocol, and one additional plant served as a control. The irrigation and drying experiments were conducted on independent groups of plants. The water content of the group (drained *Komatsuna*, 2 samples) was removed; that is, irrigation was completely stopped, whereas the other plant (control *Komatsuna*, 1 sample) was kept wet. Although the number of biological replicates was limited, an additional drained plant was measured separately and confirmed to exhibit impedance trends similar to those presented. EIS measurements were conducted every 2 h before and after draining, with appearance photographs taken at the same interval.

### Equivalent circuit design

**Fig. 2.**
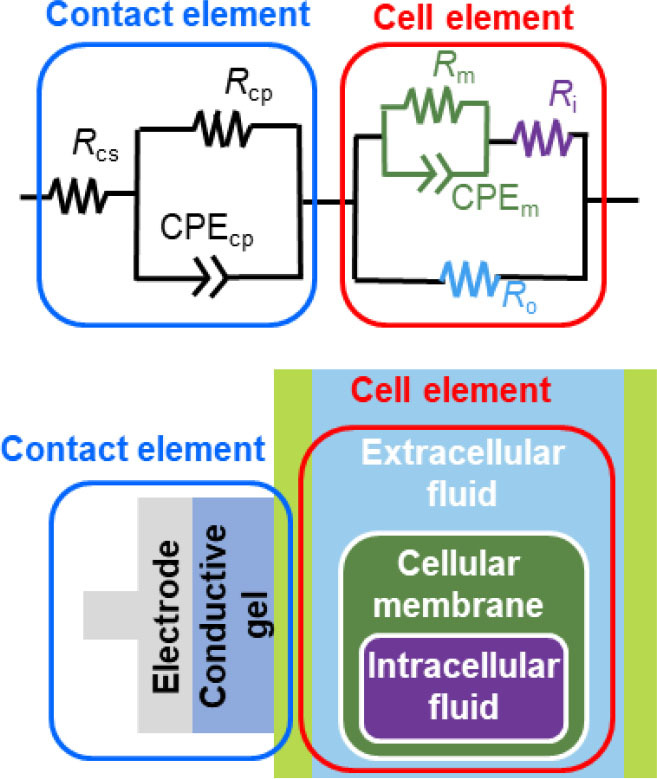
Equivalent circuit model used for analysis of EIS data obtained from plant tissues. The model consists of a contact element representing the electrode–plant interface and a cell element representing the electrical properties of plant tissues, including intracellular and extracellular fluids and membrane-associated components.

Before analyzing the experimental results, an equivalent circuit design of the EIS measurements of the cellular plant tissues was constructed based on a previous equivalent circuit design (the *Okajima model* [12]), as illustrated in Fig. 2. The purpose of this modeling was not to provide a complete physiological description, but to separate plant-related impedance components from electrode–plant interface effects in a physically reasonable manner. This design consists of a “contact element” representing the interface between the plant and the electrodes, and a “cell element” representing the plant tissue. The design replaces the capacitor connected in parallel with a contact parallel resistance (*R_cp_*) of the contact element in the Okajima model with a CPE. This modification reflects the non-ideal capacitive behavior of the interface between the petiole of the *Komatsuna* and the electrode.

The equivalent circuit of the cellular tissue, right side of Fig. 2, contains four elements: a series resistance (*R_i_*) representing the intracellular fluid, a parallel resistance (*R_m_*) representing the resistance of the cell membrane, the constant phase element (CPE) as the “capacitance-like element” of a parallel circuit, and a resistance (*R_o_*) representing the extracellular fluid in parallel with the rest of the circuit. Among these parameters, focus was placed on *R_o_* as a representative indicator of changes in extracellular conditions associated with plant water status. A CPE is an impedance component that reflects non-ideal frequency-dependent characteristics and a constant phase across an entire frequency range [9,10], and it accounts for the significant difference between standard and normal “impedance measurement” and EIS. The impedance of the CPE (Z_CPE_) is defined using the CPE index “*p*” (CPE-*p*) and the CPE constant “*T*” (CPE-*T*) as follows:

(2) $$Z_{\mathrm{CPE}}=\frac{1}{{\left(j\omega \right)}^{p}T}.$$

The EIS data were fitted using ZPlot (Scribner Inc.) to estimate the impedance parameters. All fitting procedures were performed under identical initial conditions and constraints to ensure consistency across samples and time points. The equivalent circuit model has been described in detail in our previous reports [12,13,14,15,29]; thus, only a brief outline is provided here. Several candidate configurations were evaluated in the preliminary stage, and the present model was selected based on physically consistent parameter behavior and stable convergence. The fitting uncertainties estimated by ZPlot were within 10% for the principal parameters, and no systematic deviation was observed in the residuals. Considering inherent biological variability among plants, the reported parameter values are expressed with limited significant digits to avoid over-interpretation. The parameter *R_m_* was fixed at 10^10^ Ω because preliminary fitting indicated that it assumed extremely large values and contributed negligibly within the measured frequency range. Allowing *R_m_* to vary did not significantly affect the extracted *R_o_*; therefore, fixing it improved numerical stability without altering the interpretation of the results. As shown in Fig. 2, the electrode–plant interface, including gel-related contact impedance, is explicitly incorporated into the equivalent circuit model, enabling separation of contact-related contributions from plant tissue impedance. Consequently, the extracted parameter *R_o_* is interpreted as reflecting changes in the extracellular fluid of the plant tissue rather than variations originating from the electrode–plant interface.

## Results and Discussion

**Fig. 3.**
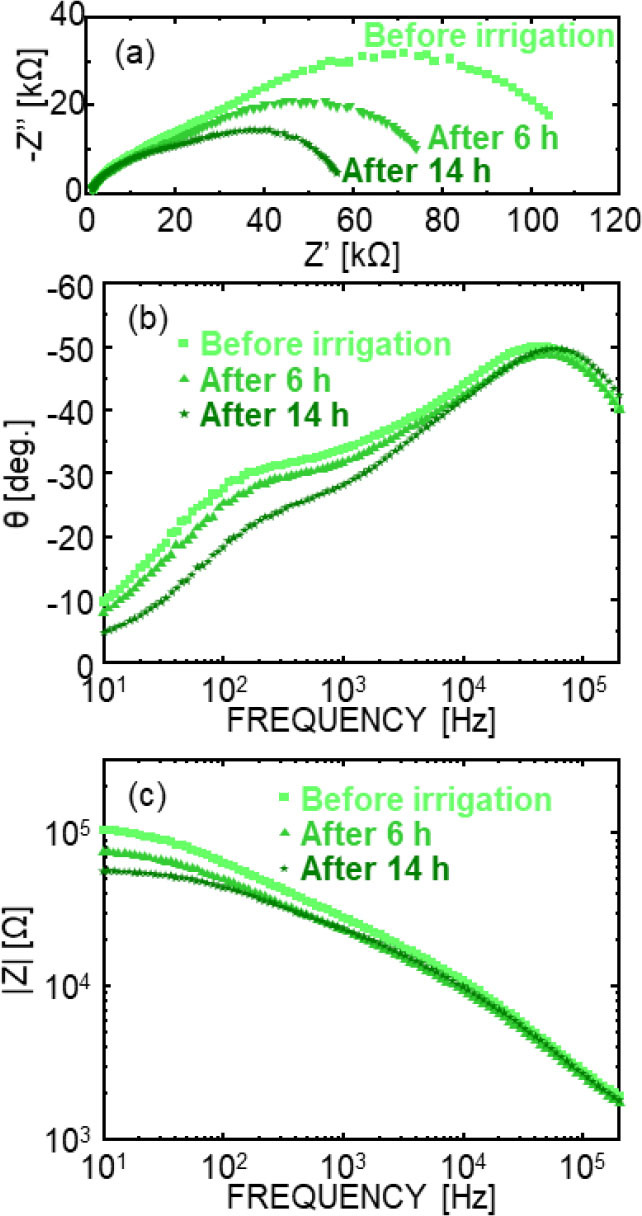
Representative impedance responses of *Komatsuna* following irrigation. (a) Nyquist plots, and Bode plots of (b) impedance magnitude |Z| and (c) phase angle θ measured at different elapsed times after irrigation. Pronounced changes were observed mainly in the low-frequency region, while the high-frequency response remained relatively stable.

First, the ability of EIS to observe biological changes in the *Komatsuna*, caused by irrigation was evaluated, since water is a key factor that affects plant growth. Given the time-resolved and measurement-intensive nature of the experiment, the following results focus on representative impedance responses. At the same time, similar temporal trends were reproducibly observed across multiple independently measured plants. Figure 3(a) shows the representative Nyquist plots of the *Komatsuna* over time after irrigation, in which the horizontal axis represents resistance and the vertical axis represents reactance. Figures 3(b) and (c) show Bode plots of the amplitude |Z| and phase θ of the petioles of *Komatsuna*, respectively.

Irrigation produced notable changes in the low-frequency regions of both plots, whereas the high-frequency regions exhibited minimal variations. In the Nyquist plot, the radius of the low-frequency semicircle decreased over time, indicating changes in the internal plant conditions, whereas the high-frequency semicircle remained stable. Similarly, in the Bode plots, |Z| decreased and θ increased in the low-frequency region, with no significant changes observed in the high-frequency region. These low-frequency-dominant variations were consistently observed across repeated measurements at each time point, suggesting that the changes reflect systematic physiological responses rather than random measurement fluctuations. These results suggest that ion fluctuations in the *Komatsuna*, due to irrigation can be observed through changes in the EIS signal, which reflect the electrical properties of plant tissues.

Because surface electrodes were used, the effective measurement depth cannot be strictly defined and likely reflects a distributed electrical pathway between the two electrodes. Therefore, the measured impedance should be interpreted as a composite response that includes tissues related to water transport rather than being attributed exclusively to specific vascular tissues. Although the precise tissue-level origin of the signal remains to be fully clarified, the reproducible changes observed under irrigation and water stress conditions suggest that physiologically relevant variations in plant water status are captured by the present measurement approach.

**Fig. 4.**
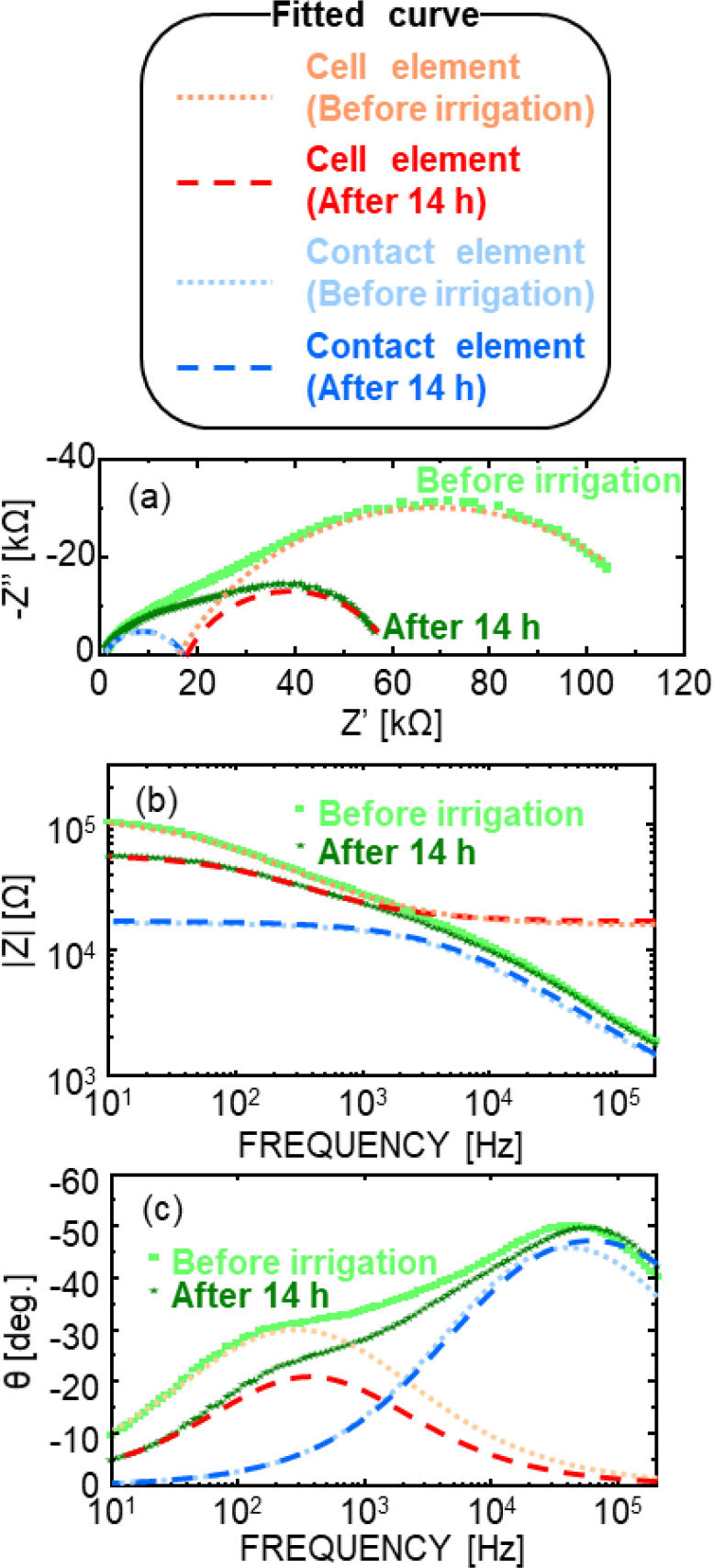
Representative impedance spectra and fitting results based on the equivalent circuit model. (a) Nyquist plots and Bode plots of (b) |Z| and (c) θ are shown together with fitted curves, illustrating the separation of the total impedance into contributions from the cell element and the contact element.

It has been reported that plants under non-water-stressed conditions exhibit lower impedance than those under water-stressed conditions [25,26], which is consistent with the results of this experiment. However, the absolute frequency range and magnitude of impedance changes are known to depend on individual plant characteristics, electrode configuration, and measurement conditions. Therefore, the present results are discussed primarily in terms of relative temporal trends. Further analysis is necessary to deconvolute the EIS signal into components such as contact properties and cellular structures, such as membranes and fluids. Because irrigation primarily alters internal conditions without significantly affecting the plant–electrode contact, the observed low-frequency impedance changes are attributed primarily to internal plant responses. Therefore, the data were fitted to the equivalent circuit model illustrated in Fig. 2, in which the cell element predominantly affected the low-frequency region and the contact element affected the high-frequency region.

Figures 4(a–c) show the Nyquist plots and Bode plots of |Z| and θ, along with the fitting results and their separation into cell and contact elements. The fitted curve is consistent with the experimental data over the entire frequency range. The fitting parameters presented here represent typical values obtained from repeated measurements, and the overall temporal trends were reproducible, although minor variations in absolute values were observed among individual plants. The estimated parameters are as follows: contact element: *R_cs_* = 10^2^ Ω, *R_cp_* = 10^4^ Ω, CPE-*p* = 0.7, CPE-*T* = 10^−8^; cell element: *R_o_* = 10^4^–10^5^ Ω, *R_i_* = 10^2^ Ω, CPE-*p* = 0.7, CPE-*T* = 10^−7^. *R_m_*, which represents a small current through the cell membranes, was fixed at *R_m_* = 10^10^ Ω, which is three orders of magnitude larger than *R_o_*. The fitting results shown in Figs 4(a–c) correspond to a representative impedance spectrum and are not averaged values. Considering the variability observed among individual plants, the reported parameter values are presented with limited significant digits to avoid overinterpretation of fitting precision. Changes were observed in the cell element over time after irrigation, whereas the contact element remained constant within the experimental uncertainty. This separation supports the validity of the equivalent circuit approach for distinguishing plant-related impedance changes from those originating at the electrode–plant interface. These results indicate that the fitting analysis can reasonably isolate changes associated with plant water conditions, even in noninvasive surface measurements.

**Fig. 5.**
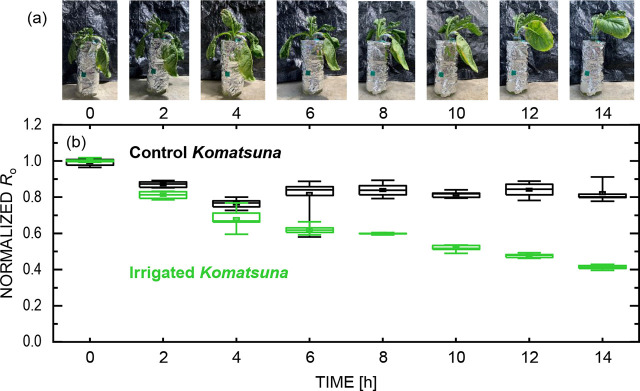
Physiological and electrical responses of *Komatsuna* during irrigation. (a) Time-dependent changes in the visual appearance of irrigated plants. (b) Normalized *R_o_* values derived from equivalent circuit fitting as a function of elapsed time after irrigation, shown for irrigated and control conditions.

Next, the changes in the separated cell element and physiological conditions of the *Komatsuna* are discussed. Figure 5(a) shows the changes in the appearance of the irrigated *Komatsuna*. After irrigation, the *Komatsuna* expanded and recovered. In addition, the water content increased (from 81.7 % to 87.2 % after 14 h), whereas the control group exhibited only a minor change (from 77.9 % to 78.4 %). Although the number of plants was limited, these trends were consistently observed across multiple measurements, indicating that irrigation effectively restored plant water status. Figure 5(b) shows the normalized *R_o_* values before and after irrigation. *R_o_*, which represents the resistance of the extracellular fluid, decreased in both groups, though with different temporal behaviors. In the control group, *R_o_* decreased until 4 h and then stabilized. In contrast, the irrigated group exhibited a more rapid initial decrease, followed by a continued gradual decline over 6–14 h. The use of normalized *R_o_* emphasizes relative changes over time and allows comparison among plants with different absolute impedance values. Before irrigation, the extracellular fluid likely had a relatively high ion concentration owing to the dry conditions, leading to reduced effective conductivity. Irrigation diluted the extracellular fluid, thereby increasing conductivity and decreasing *R_o_* [30]. These observations suggest that *R_o_* can serve as a sensitive indicator of changes in plant water status, even when absolute impedance values vary among individual plants.

**Fig. 6.**
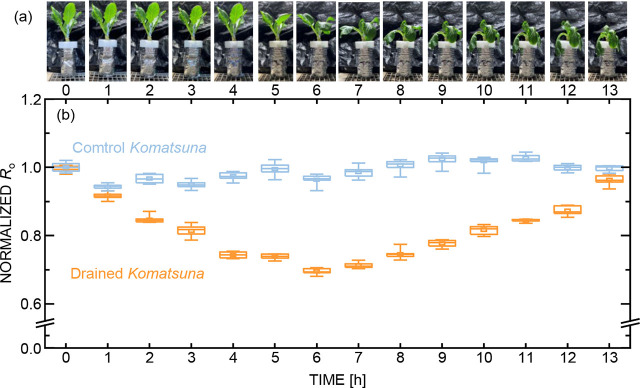
Physiological and electrical responses of *Komatsuna* during drying. (a) Time-dependent changes in the visual appearance of drained plants. (b) Normalized *R_o_* values as a function of elapsed time after drying for drained and control conditions, highlighting impedance changes preceding and exceeding visually detectable stress.

Accordingly, monitoring plant water stress is crucial for agricultural management. The feasibility of using EIS for *in situ* observation of biological changes in *Komatsuna* under drying-induced water stress was further examined. Figure 6(a) shows the appearance of the drained *Komatsuna*, whereas Fig. 6(b) depicts the temporal changes in *R_o_* for both the drained and control *Komatsuna*. Representative results are shown, while similar qualitative trends were confirmed in an additional drained plant. The parameters were calculated using a similar approach as in the irrigation experiment (contact element: *R_cs_* = 10^2^ Ω, *R_cp_* = 10^3^ Ω, CPE-*p* = 0.6–0.9, CPE-*T* = 10^−8^–10^−7^; cell element: *R_o_* = 10^4^ Ω, *R_i_* = 10^2^ Ω, CPE-*p* = 0.5–0.6, CPE-*T* = 10^−8^–10^−7^). The *R_o_* of the control *Komatsuna* remained nearly constant, whereas the drained *Komatsuna* exhibited a progressive change in *R_o_* and physiological condition. In the early stage (up to 4 h after drainage), there was no visible change in appearance; however, *R_o_* decreased. This behavior is plausibly attributed to an increase in extracellular ion concentration resulting from initial water loss, thereby enhancing conductivity [31]. This suggests that EIS can detect early water stress that is not visually apparent. In the mid-stage (between 5 and 8 h), the *Komatsuna* began to wilt, indicating visible water stress due to reduced turgor pressure [32]. During this stage, the trend of *R_o_* reversed and began to increase. Such a reversal may reflect changes in ion mobility and water availability in the extracellular space rather than a simple monotonic response [33,34]. In the late stage (from 9 to 13 h), the *Komatsuna* completely wilted, with no further visible changes, whereas *R_o_* continued to increase. This continued change suggests that EIS can capture ongoing physiological stress even after macroscopic symptoms saturate.

Taken together, the present results demonstrate that electrochemical impedance spectroscopy provides access to dynamic, tissue-level physiological responses of plants under changing water conditions. By separating cell-related and contact-related contributions through equivalent circuit analysis, the observed impedance variations can be interpreted in terms of water- and ion-dependent processes occurring within the extracellular and intracellular compartments. In particular, the temporal evolution of *R_o_* consistently reflected changes in plant water status under both irrigation and drying, often preceding or extending beyond visible morphological responses. The inter-plant variability of the fitted parameters is summarized in Figs. 5 and 6 using box-and-whisker plots, where comparable degrees of dispersion were observed across experimental conditions. The normalized *R_o_* values were calculated using the median value at 0 h for each dataset; because the mean and median at 0 h were nearly identical, this procedure does not affect the interpretation of the temporal trends. Normalization was adopted to emphasize systematic changes associated with irrigation and water stress, given the inherent inter-individual variability in the raw impedance values. Figures 5 and 6, therefore, summarize parameter distributions across individual plants rather than a single representative sample. These findings support the concept that EIS enables *in situ*, non-invasive detection of early water stress by capturing subtle electrical signatures associated with cellular-scale physiological regulation. Such sensitivity is particularly relevant for plant ecophysiology and agricultural monitoring, where early stress detection is critical but often limited by reliance on visual or destructive measurements. Although the present study is based on a limited number of plants and emphasizes qualitative trends rather than statistical generalization, and no statistically rigorous correlation analysis between *R_o_* and water content is claimed, provides an initial physiological framework linking impedance parameters to plant water status, providing a foundation for future quantitative validation, species comparison, and integration with conventional physiological indices.

## Conclusion

This preliminary study examined changes in plant water status in *Komatsuna* during irrigation and drying processes using EIS combined with an equivalent circuit analysis. The results suggest that EIS can sensitively capture dynamic physiological responses associated with water movement within plant tissues. Irrigation produced noticeable impedance variations mainly in the low-frequency region. In contrast, the high-frequency region remained relatively stable, indicating that the observed responses predominantly originate from tissue-level electrical properties rather than electrode-related effects. Fitting analysis further confirmed that these changes were associated primarily with cell-related elements, with minimal contribution from the electrode–plant interface. During recovery from dryness through irrigation, the extracellular resistance *R_o_* exhibited a temporal behavior distinct from that of the control group, suggesting a close relationship between *R_o_* and water uptake or redistribution within the tissue. Under drying conditions, *R_o_* responded more rapidly than visible morphological changes and continued to vary even after no further visual symptoms were observed. This highlights the capability of EIS to detect early physiological alterations related to water stress that are not accessible through visual inspection alone. Overall, these findings indicate that EIS represents a promising non-invasive tool for early, *in situ* detection of plant water stress, providing insight into cellular-scale physiological changes underlying macroscopic plant responses. Although this study involved a limited number of plants due to experimental constraints, it serves as a proof of concept for EIS-based monitoring of plant water status. Future work incorporating larger sample sizes and broader environmental conditions will be necessary to further validate and biologically contextualize the extracted impedance parameters.
